# Post-diagnosis body mass index and mortality among women diagnosed with endometrial cancer: Results from the Women’s Health Initiative

**DOI:** 10.1371/journal.pone.0171250

**Published:** 2017-02-02

**Authors:** Hannah Arem, Ruth M. Pfeiffer, Steven C. Moore, Melinda L. Irwin, Michael J. LaMonte, Gloria E. Sarto, Rami Nassir, Juhua Luo, Rowan T. Chlebowski, Louise A. Brinton, Charles E. Matthews

**Affiliations:** 1 George Washington University Milken Institute School of Public Health, Washington, DC, United States of America; 2 Division of Cancer Epidemiology and Genetics, National Cancer Institute, Bethesda, MD, United States of America; 3 Yale School of Public Health; Yale Cancer Center, New Haven, CT, United States of America; 4 School of Public Health and Health Professions, Department of Epidemiology and Environmental Health, University at Buffalo, Buffalo, NY, United States of America; 5 UW Madison Department of Obstetrics and Gynecology, Madison, WI, United States of America; 6 Department of Biochemistry and Molecular Medicine, University of California, Davis, CA, United States of America; 7 Department of Epidemiology and Biostatistics, School of Public Health–Bloomington, Indiana University, IN, United States of America; 8 Los Angeles Biomedical Research Institute at the Harbor-UCLA Medical Center, Torrance, CA, United States of America; Universite du Quebec a Trois-Rivieres, CANADA

## Abstract

Higher body mass index (BMI) measured before endometrial cancer diagnosis has been associated with greater risk of developing endometrial cancer and higher mortality, but the association between BMI measured after diagnosis and mortality risk is unclear. We identified 467 women (91 deaths) in the Women’s Health Initiative (WHI) with information on BMI measured after diagnosis and used Cox proportional hazards regression to generate hazard ratios (HR) and 95% confidence intervals (CI) for all-cause mortality. Comparing BMI 35+ with <25 kg/m^2^, we observed no association with all-cause mortality (HR = 1.02, 95% CI 0.55–1.91). Our study does not support the hypothesis that higher BMI after endometrial cancer diagnosis is associated with poorer survival.

## Background

There is consistent evidence that higher body mass index (BMI) is associated with higher risk of incident endometrial cancer [1]; each 5-unit BMI increase has been associated with a 59% increased risk of developing endometrial cancer [2]. Published studies of BMI measured *before* endometrial cancer diagnosis and all-cause mortality showed an approximate 2-fold increase in both all-cause and endometrial cancer specific mortality [3, 4].

While observational studies of BMI measured *after diagnosis* and mortality among breast cancer survivors (another BMI-related cancer) have suggested worse survival with higher BMI [5], evidence on BMI measured *after diagnosis* and mortality among women diagnosed with endometrial cancer is limited. An estimated 70% of endometrial cancer survivors are obese [6], constituting a potential public health target for weight loss. In the present study we draw on data from the Women’s Health Initiative (WHI) Clinical Trial and Observational Study to confirm our recent findings in the US National Institutes of Health (NIH)-AARP cohort [7] that higher BMI measured after diagnosis was associated with greater mortality risk among endometrial cancer survivors.

## Materials and methods

Eligibility criteria and recruitment methods for WHI have been published [8]. In short, 161,808 women were enrolled in the WHI between October 1993 and December 1998 from 40 US clinical centers. Eligible women were between 50 and 79 years of age, postmenopausal, had an anticipated survival of >3 years and were accessible for follow-up. Of the 161,808 women enrolled in WHI, 1,549 were diagnosed with invasive endometrial cancer before September 2013. We define cancer survivors from the moment of diagnosis, in accordance with guidelines from the Institute of Medicine, the American Society for Clinical Oncology, and other professional societies and government entities [9]. We excluded women who reported a hysterectomy before baseline (n = 20) or prior cancer before baseline visit (n = 114). We also excluded those with stage 4 disease (n = 77) because it is unlikely that BMI would affect prognosis for metastasized disease. According to study protocol, BMI data was collected at years 3 and 6 post baseline; of the remaining 1,338 women, 467 women had data on BMI collected after diagnosis. Women diagnosed after year six were not eligible for this analysis and we used the BMI measure closest to diagnosis (median 1.4 years) in the present analysis.

All participants provided written informed consent. Institutional review board approval was obtained from each of the participating study centers and from the Fred Hutchinson Cancer Research Center, which currently serves as the IRB of record for the WHI. In the WHI there were 40 clinical site IRBs, the coordinating center IRB, and ethical review at NIH; participants completed informed consent forms approved by local institutional review boards. The funders had no role in study design, data collection and analysis, decision to publish, or preparation of the manuscript. More information about proposing a paper using WHI data can be found online at https://www.whi.org/researchers/SitePages/Home.aspx and proposals can be submitted to p&p@whi.org

We categorized BMI as normal weight, overweight, class 1 obese, and class 2 obese (18.5-<25 (referent), 25-<30, 30-<35, 35+ kg/m^2^). The correlation between pre and post-diagnosis BMI was r = 0.95. In the study population 40 women lost more than three BMI units (kg/m^2^), 398 women had no change within 3 units, and 29 women gained three or more BMI units. We used IVEware 2.0 (Ann Arbor, MI, 2002) to impute values for missing variables (<5% missing for all categories), using 10 iterations and five imputations. We used Cox proportional hazards models to estimate hazard ratios (HR) and 95% confidence intervals (CI) in SAS version 9.2 (SAS Institute Inc, Cary NC). Proc mianalyze was used to combine results from the five imputed datasets. The underlying time metric was calculated from post-diagnosis questionnaire to death or end of follow-up (through September, 2013) whichever occurred first. We evaluated the proportional hazards assumption by modeling interaction terms of the continuous main exposure with follow-up time for each model, and found no violation of the model assumptions.

We first built a model adjusted only for age. We added tumor grade and stage to models as they are strongly associated with survival, and then tested the variables that showed significant differences by BMI category in Table 1 as confounders and eliminated those that did not change BMI parameter estimates by >10%. Thus, our final model included age at diagnosis, tumor stage and grade, diabetes and age at menarche. We added self-reported health status in sensitivity analyses to allay concerns that health status is in the causal pathway between BMI and mortality. Health status is a strong independent predictor of mortality and it is possible that higher BMI may lead to health complications that increase risk of mortality. Neither adding trial arm (dietary modification, menopausal hormone therapy, observational study) to the models, nor stratifying by trial arm changed estimates.

**Table 1:** Baseline characteristics of the women diagnosed with endometrial cancer in the Women’s Health Initiative study populations by level of baseline body mass index (BMI) (kg/m^2^) (N = 467).

Body Mass Index (BMI)	18.5-<25		25-<30		30-<35		35+		p-value
Deaths/N	28/127		20/138		22/110		21/92		0.334
Age at diagnosis, years (mean, sd)	67.1	0.7	67.1	0.6	66.6	0.6	65.2	0.6	<0.001
	**N**	**%**	**N**	**%**	**N**	**%**	**N**	**%**	
**Tumor summary stage**									0.758
Localized	113	89.0	125	90.6	97	88.2	85	92.4	
Regional/Distant	14	11.0	13	9.4	13	11.8	7	7.6	
**Tumor grade**									0.717
Well differentiated	41	32.3	39	28.3	39	35.5	30	32.6	
Moderately differentiated	49	38.6	67	48.6	41	37.3	41	44.6	
Poorly differentiated	24	18.9	22	15.9	20	18.2	17	18.5	
Anaplastic grade 3	12	9.5	8	5.8	7	6.4	3	3.3	
**Education**									0.013
<High school/ high school graduate	25	19.7	27	19.6	34	30.9	20	21.7	
Post high school/ some college	32	25.2	36	26.1	37	33.6	37	40.2	
College or graduate degree	70	55.1	73	52.9	39	35.5	34	37.0	
**Race/ethnicity**									
Non-Hispanic White	114	89.8	130	94.2	104	94.6	76	82.6	<0.001
African American	2	1.6	5	3.6	3	2.7	14	15.2	
Other	10	7.9	3	2.2	3	2.7	2	2.2	
**Self-reported diabetes**									0.003
No	124	97.6	134	97.1	102	92.7	80	87.0	
Yes	3	2.4	4	2.9	8	7.3	12	13.0	
**Age at menarche**									0.008
≤12 yrs old	53	41.7	75	54.4	63	57.3	59	64.1	
13+ yrs old	74	58.3	63	45.7	47	42.7	33	35.9	
**Parity**									<0.001
Nulliparous	20	15.8	25	18.1	7	6.4	21	22.8	
1–2	67	52.8	37	26.8	43	39.1	31	33.7	
3–4	33	26.0	60	43.5	40	36.4	28	30.4	
5+	7	5.5	14	10.1	20	18.2	12	13.0	
**Age at menopause**									<0.001
<50	45	40.2	41	36.3	38	38.8	15	19.5	
50-<55	37	33.0	43	38.1	35	35.7	27	35.1	
55+	28	25.0	29	25.7	18	18.4	26	33.8	
**Oral contraceptive use**									0.063
Never	73	57.5	97	70.3	61	55.5	59	64.1	
Ever	54	42.5	41	29.7	49	44.6	33	35.9	
**Hormone use at baseline**									<0.001
Never	22	17.3	39	28.3	26	23.6	57	62.0	
Former	16	12.6	37	26.8	28	25.5	22	23.9	
Current	89	70.1	59	42.8	53	48.2	13	14.1	
**Smoke**									0.601
Never	70	55.1	81	58.7	58	52.7	53	57.6	
Former	52	40.9	53	38.4	45	40.9	33	35.9	
Current	5	3.9	3	2.2	5	4.6	6	6.5	
**Self-reported health**									<0.001
Excellent/very good	98	77.2	91	65.9	68	61.8	36	39.1	
Good	24	18.9	40	29.0	39	35.5	41	44.6	
Fair/Poor	5	3.9	7	5.1	3	2.7	15	16.3	
**Marital Status**									0.181
Married or living as married	78	61.4	84	60.9	80	72.7	62	67.4	
Not married	49	38.6	54	39.1	30	27.3	30	32.6	
**Randomized to HRT Arm**	8	6.3	18	13.0	23	20.9	11	12.0	0.010
**Randomized to DM Arm**	53	41.7	86	62.3	65	59.1	58	63.0	0.002
**Observational Study**	68	53.5	42	30.4	32	29.1	26	28.3	<0.001

Abbreviations: SD: standard deviation, HRT: Hormone replacement therapy; DM: dietary modification

Column percentages may not total to 100.0 due to missing data.

P-values were calculated using the t-test for continuous variables and the chi-squared test for categorical variables.

## Results

With a median 10.2 years of follow-up, we observed 91 all-cause deaths. Women with a BMI 35+ kg/m^2^ were slightly younger, less educated, more likely to be African American and to have diabetes. These class 2 obese women also tended to have a younger age at menarche, older age at menopause, report never menopausal hormone therapy use, and report worse self-reported health (Table 1).

Before adjusting for co-variables, the log-rank test demonstrated a non-significant p-value = 0.364. In multivariate models, BMI was not associated with mortality when comparing BMI 35+ to <25 kg/m^2^ (HR = 1.02, 95% CI 0.55–1.91) (Table 2). The addition of self-reported health status to the models also yielded null results (HR = 0.87, 95% CI 0.45–1.69). We did not observe differences in the association between BMI and mortality when stratifying by the median 1.4 years from diagnosis to questionnaire (HR_<1.4 years_ = 0.99, 95% CI 0.75–1.31; HR_≥1.4 years_ = 0.99, 95% CI 0.72–1.35). Adding additional demographic factors as described in Table 1 did not change interpretation of results.

**Table 2:** Hazard ratios and 95% confidence intervals for body mass index (BMI) and mortality risk among endometrial cancer survivors in the WHI (n = 467).

	Deaths/Total N	Model 1 HR (95% CI)	Model 2 HR (95% CI)	Model 3 HR (95% CI)
**BMI, kg/m**^**2**^				
18.5-<25	28/127	1.00	1.00	1.00
25-<30	20/138	0.62 (0.35, 1.10)	0.67 (0.37, 1.21)	0.64 (0.36, 1.16)
30-<35	22/110	0.85 (0.48, 1.48)	0.82 (0.46, 1.45)	0.74 (0.41, 1.34)
35+	21/92	1.22 (0.68, 2.17)	1.02 (0.55, 1.91)	0.87 (0.45, 1.69)
*P-trend*		0.476	0.919	0.693

Model 1 was adjusted for age at diagnosis only.

Model 2 was additionally adjusted for tumor stage, tumor grade, diabetes and age at menarche.

Model 3 was adjusted for all of the above factors, in addition to health status.

## Discussion

Our results do not support an association between BMI measured after diagnosis and mortality among endometrial cancer survivors. These findings are in contrast with another study of post-diagnosis BMI and mortality in the NIH-AARP Diet and Health Study, which found an approximate 2-fold increase in mortality risk comparing women with a BMI 35+ to normal weight women (<25 kg/m^2^) [7]. One reason for these discrepant findings may be due to differences in the timing of BMI measurement between studies, as BMI was measured a median of 1.4 years after diagnosis in WHI and median 4.1 years after diagnosis in NIH-AARP. In this WHI study we did not observe effect modification by time from diagnosis to BMI measurement. While the available metrics the WHI and NIH-AARP populations appeared similar, it is possible that there were differences in participant characteristics that we were unable to account for in this analysis. Alternatively, our results may simply show that there is no association between post-diagnosis BMI and mortality. Another study of endometrial cancer patients who participated in a randomized surgery and adjuvant radiation therapy found that among 380 patients, morbidly obese patients (BMI≥40 kg/m^2^) had a nearly threefold increased risk of death compared to BMI <30 (HR = 2.77; 95% CI 1.21–6.36); this increased mortality risk was not apparent among patients with a BMI of 30–39.9 [10]. This finding suggests there may be a BMI threshold beyond which mortality risk is escalated; still, while there was an association with all-cause mortality, there was no association with endometrial cancer recurrence.

A recent meta-analysis among endometrial cancer survivors showed a dose–response curve between BMI and all-cause mortality, with a 66% increased risk of death comparing women with a BMI 40+ to <25 kg/m^2^ [11]. The timing of BMI measurement in relation to diagnosis varied in this published meta-analysis. In a previous analysis of endometrial cancer survivors in WHI, pre-diagnosis BMI measured a median of 5.1 years before diagnosis was associated with an all-cause mortality risk HR = 1.85 (95% CI 1.19–2.88) comparing women with a BMI ≥ 35 kg/m^2^ to women with BMI < 25 kg/m^2^ [4]. It is unclear why we found an association between pre-diagnosis BMI and mortality in this cohort but not post-diagnosis BMI. In future studies it could be important to account for intentional versus unintentional weight loss, or change in weight from pre to post-diagnosis.

In our previous analysis of pre-diagnosis BMI in WHI, the magnitude of association was stronger for BMI and endometrial-cancer specific mortality (HR = 2.23, 95% CI 1.09–4.54). This association was previously measured in the Cancer Prevention Study II, where there was over a six-fold association between pre-diagnosis BMI 40+ and normal BMI women for endometrial cancer-specific mortality [12]. In the present post-diagnosis cohort we were unable to assess cause-specific mortality because of insufficient cause-specific deaths. We were not able to assess associations separately for women with a BMI over 40 because of insufficient numbers in this category.

Limitations of our study include the lack of data on treatment, which may in turn affect survival. However, cancer stage (which we included in our adjusted models) has been shown to be the strongest predictor of prognosis among women with endometrial cancer [13]. We performed a post-hoc power calculation, whereby for BMI, we had 81% power to detect a HR of 2.23 (the HR for pre-diagnosis BMI and mortality in this WHI cohort) comparing the highest to the lowest BMI category using a type I error of p = 0.05 (Power 3.0, NCI). We also did not have enough deaths to perform sub-analyses by cause of death. We were unable to adjust for physical activity as this data was available for only 158 women. Although we had information on some comorbidities, there may be unmeasured factors confounding the association between BMI and mortality that we were unable to account for in this analysis. Selection bias may have influenced results, as the individuals who had later stage disease may have died before follow-up. Strengths of the study include the geographic diversity of the cohort, the centralized adjudicated outcomes, and the multiple time points of collection on BMI data, which allowed us to examine BMI recorded after cancer diagnosis.

While obesity has been recognized as a public health threat in the general population [14], research focused on cancer survivors may present key opportunities for targeted interventions, as cancer diagnosis has been cited as a “teachable moment” for lifestyle change [15]. Future research should explore the effects of change in post-diagnosis BMI on cancer outcomes including quality of life, fatigue, and biomarkers of recurrence in addition to survival.

In summary, the present findings from the WHI do not support the hypothesis that BMI measured after diagnosis is associated with survival among women with endometrial cancer. Additional prospective data from larger cohorts with greater variation in timing and frequency of post diagnosis BMI assessment are needed to further clarify this association that has important implications to public health and women’s healthcare in an aging population.
